# Catalogue of Such British Plants as Have Been Found in Any Shape Serviceable to Man, Whether in a Medicinal, Œconomical, Culinary, or Agricultural Point of View, Together with an Account of the Uses Which They Have Been Made to Answer, and an Accurate Botanical Description of Each Plant

**Published:** 1804-05-01

**Authors:** 


*Catalogue of such British Plants as have been found in any Shape serviceable to Man, whether in a medicinal, œconomical, culinary, or agricultural Point of View, together with an Account of the Uses which they have been made to answer, and an accurate Botanical Description of each Plant.*

( Continued from our last, pp. 368—372. )

5. PINGUICULA. *P. vulgaris. P. Gesneri.*

*Ang.* Butterwort. Yorkshire sanicle.

*Gen. Desc.* *Blos.* gaping, ending in a spur. *Cal.* two-lipped, five-cleft. *Caps.* one cell.

*Spec. Desc.* Nectary cylindrical, as long as the petal. *Leaves* covered with soft prickles, secreting a glutinous liquor. *Blossom* violet, purple and reddish, and an ash-coloured woolly shot on the palate. *On bogs.* May.

*Use.* The juice of the leaves kills lice; and the common people use it to cure cracks or chaps in cows udders. It is made much use of by the inhabitants in the north of Sweden; the fresh gathered leaves are put into a filter or strainer, through which is poured milk warm from the rein deer, and the milk being sat by for a day or two, to become acescent, it acquires consistence and tenacity; the whey does not separate, nor does the cream, and in this state it is an extremely grateful article of food. Half a spoonful of this prepared milk, mixed with fresh warm milk, converts the latter into its own nature; and this again will have the same effect, and so on, without having recourse a second time to the leaves—*Linn.* It did not succeed with cow's milk—*Mr. Hawkes.* This plant is generally supposed to be injurious to sheep, and is imagined to occasion a disease which farmers call the rot. But it may be a question, whether the rot in sheep be so much owing to the vegetables in marshy grounds, as to a flat insect, called a fluhe *(fasciola hepatica)* which is found in these wet situations adhering to the stones and plants, and likewise in the livers and biliary ducts of sheep afflicted with the rot. From experiments made on purpose, and conducted with accuracy, it appears, that neither *sheep*, cows, horses, goats, nor swine will eat this plant—*Withering.*

6. Lycopus. *L. Europæus. L. palustris, pseudo marrubium palustre.*

*Ang.* Water hore-hound, hore-hound gypsey-wort.

*Gen. Desc. Bloss.* four-cleft; one segment notched at the end; *stamens* distant; *seeds* four, blunt.

*Spec. Desc. Leaves* indented, serrated; a little hairy. *Stem* with four corners and four hollow sides, hairyish. *Blossom* whitish with a tinge of purple, hairyish within, upper segment slightly notched, lower one with purplish spots at the inside; between the stamens frequently two or three shorter filaments, generally without anthers. *Germen* on a yellow glandular receptacle. *Flowers* several together, in the bosoms of the upper leaves. *Branches* opposite, rising from the bosom of the leaves. *In sandy ground on the banks of streams and ponds.* *Bloss.* July, Sept.

*Use.* It dyes black, and has been used with linen and clothes. The juice gives a permanent colour to linen, wool, and silk, which will not wash out. Travelling gypsies use it to stain their faces. Sheep and goats eat it; cows and horses refuse it

7. Orchis. *O. mascula, satyrion in Pharm. Ed. Cynosorchis moriomas.*

*Ang.* Early orchis, male fool-stones.

*Gen. Desc.* Bloss. gaping; nectary like a horn behind the flower.

*Spec.*

*Spec. Desc.* Lip of the nectary four-lobed, finely scolloped, dotted at the base; horn blunt; upper petals turned back. Bulbs oval, entire, fixed to the base of the stem. *Leaves* spear shaped, bright green, and shining above, sea-green underneath, with longitudinal parallel veins, usually with dark brown spots: Floral leaves longer than the germen. *Flowers* purple, rarely white, numerous, the outer petals bent back, not at all approaching the helmet, rather pointed; the middle one expanding and lying over the two lower ones, which approach closely and are of a paler colour. *Stem,* twelve to fifteen inches high. *In meadows and pastures.* Bloss. May.

*Use.* This plant has a place in the materia medica of the Edinburgh Pharmacopœia, on account of the root, which abounds with glutinous slimy juice of a sweetish taste: To the smell, faint and somewhat unpleasant. This mucilaginous quality of the root has recommended it as a *demulcent,* and it has been generally employed with the same intentions and in the same complaints as the root of althæa and gum arabic—*Woodville.* Of the root of this species of orchis it is now known that salep is made, which has been hitherto imported from Turkey, and from the East, at a considerable expence. Dr. Percival has published, on the authority of Mr. Moult, of Rochdale, the best method of curing the orchis root; and recommends the process from his own knowledge of its success, adding that he had seen many specimens of salep thus prepared, equal, if not superior, to any imported from the Levant. The root should be gathered when the seed is formed, and the stalk is about to fall, for then the new bulb, of which the salep is made, is arrived at its full size, and may be distinguished from the old one by a white bud rising on the top of it. The new root is to be washed in water, and the fine brown outer skin taken off by means of a small brush, or by dipping in hot water and rubbing it with a coarse linen cloth. The roots thus cleaned are to be spread on a tin plate, and placed in an oven heated to the degree of a bread oven, where, in six or ten minutes, they will have acquired a transparency like horn, without any diminution of bulk. In this state they are to be removed to harden and dry in the air, which will require some days to effect; or by a very gentle heat they may be finished in a few hours—*Percival.* As an article of diet, salep is accounted extremely nutritious, and has been thought fit to constitute a part of the provisions of every ship's company, to prevent famine at sea. An ounce of this article, together with the same same quantity of portable soup *(the dried gelatinous part of flesh),* in two quarts of boiling water, will afford a man sufficient subsistence for a day—*Woodville.* Several authors have recommended the use of salep in *diarrhœa, dysentery, dysury,* and *calculous* complaints; but in the symptomatic fever, which arises from the absorption of pus, from ulcers in the lungs, from wounds, or from amputations, salep is an admirable demulcent, and well adapted to resist that dissolution of the crasis of the blood, which is so evident in these cases—*Percival.* Salep, prepared as above, may be sold for less than a shilling a pound; and it is to be hoped we shall no longer be supplied, at a high price, from foreign markets, with an article that our own country can supply in almost any quantity.

Mr. Mault made his experiments on the roots of the *O. Mascula* only; but those of the *O. Morio* are equally proper for the purpose, and it is probable that every species of the plant may be used indiscriminately—*Withering.* It must be propagated by the roots, for the seeds seldom come to perfection. These roots have been noticed ever since the time of Dioscorides for their supposed aphrodisiac qualities.

8. Salix. S. triandra.

*Ang.* Smooth willow.

*Gen. Desç.* *Catkin,* each scale containing one flower. Bloss. none. *Male,* a nectariferous gland at the bottom of the flower.

*Female.* Style cloven; caps. one cell; two valves; seeds downy.

*Spec Desc.* *Leaves* oblong, spear-shaped, tapering; the lower ones dark green, paler underneath, serrated, one to three inches long, on leaf stalks. *Flowers* with three stamens, sometimes two. Male catkins conical, changing to cylindrical, upright, one to one and a half inch long; fruit stalks half an inch long; nectaries 2. Fem. catkin slender, upright; fruit stalks one inch long. *Stem* six feet high and more, smooth, yellowish green, branching; upper branches shortest, often spotted with red. The female plant is rare. *Woods, hedges, or banks of rivers.* Bloss. April.

*Use.* The bark, in doses of one or two drachms, has been found efficacious in curing the ague, as a substitute for Peruvian bark.

9. Salix. *S. pentandra.* *S. hermaphroditica.*

*Ang.* Sweet willow. Bay leaved willow.

*Gen. Desc.* *As above.*

*Spec.*

*Spec. Desc.* Leaves egg shaped, acute, yellowish green, pour out at the edges a yellow gum from each tooth; glossy; in hot weather exhale an odoriferous perfume; eleven on barren, six or eight on fertile shoots. *Flowers* with five stamens. Catkins very yellow, sweet scented. *Nectaries* three, sometimes wanting at the extremity of the catkin, and supported by three perfect stamens. Stem six, ten, or twelve feet high; branches yellowish purple. *Woods and Hedges.* Bloss. April.

*Use.* The bark is an astringent, and has been used as a substitute for Peruvian bark. The leaves dried afford a good yellow dye. The branches are cut to make springles: it is much used in Yorkshire for the largest baskets. Sheep and goats eat it. The wood crackles in the fire.

10. Salix. *S. fragilis.* *S. folio longo latoque splendente.*
*Ang.* Crack willow.
*Gen. Desc.* *As above.*
*Spec. Desc.* *Leaves* egg-spear-shaped; leaf-stalks toothed with glands. A tall tree. Bark wrinkled, grey. The branches, struck with the finger, break off at the shoot of the present year. *Woods, hedges, river-banks.* Bloss. April, May.

*Use.* The bark is astringent and bitter; hence it has been thought a good substitute for Peruvian bark, and has been found to stop the paroxysms of *intermittents*; it is likewise recommended in other cases requiring *tonic* or astringent remedies, and has been admitted into the Edinburgh Materia Medica. The bark of some other species of salix possess similar qualities, but that of S. triandria is the most effectual of any of this genus.—*Woodville.* The bark, in doses of one or two drachms, will cure agues.—*Med. Com.* It thrives in any soil if sufficiently moist, grows quick, and bears cropping.

11. Salix. *S. alba.*
*Ang.* Common willow. White willow.
*Gen. Desc.* *As above.*
*Spec. Desc.* Leaves spear shaped, tapering to a point, sharply and elegantly serrated, shining but pubescent above, white and silky underneath.

M. catkins cylindrical, blunt, one inch and a half to two inches long, on fruit stalks half an inch long.

F. cat. slender, two inches long, fruit stalk one inch, stamens two, nectaries two. A tall straight tree this, with the s. fragilis, being the largest trees of the kind; the bark grey, cracked; branches numerous, upright, expanding, grey, grey, or brown green. Inner bark green. *Woods, hedges, and wet pastures.* Bloss. April.

*Use.* An account of the efficacy of the bark of this tree in curing intermitting fevers, has been given by the Rev. Mr. Stone. He gathers the bark in summer, when full of sap, dries it by a gentle heat, and gives a drachm powdered, every four hours between the fits. In a few obstinate cases he mixed one-fifth of Peruvian bark, *Phil. Trans. liii. p.* 195. It is remarkable that in wet situations, where intermittents are most prevalent, this tree grows naturally. From the present high price of Peruvian bark, and the scanty supply of that article from S. America, hardly equal to the consumption, the *white* willow bark is likely to become an object worth the attention of physicians, if its success upon a more enlarged scale becomes equal to Mr. S's experiments, to whom the world will be much indebted for the communication. The bark of other species having the same properties, it would be useful to ascertain by experiment, which should be preferred. This bark will tan leather. Horses, cows, sheep, and goats eat the leaves and the young shoots. Bees are very fond of the flowers.—*Withering.*

The bark will dye yarn of a cinnamon colour ; and is of so *astringent* a quality that one scruple has been of much service in intermittents. A decoction of it, used as a bath, is affirmed by Haller, from his own experience, to have been very beneficial to ricketty children. The inner bark is used in Kamschatka as a miserable substitute for bread. From the catkins of every species of salix, which has fragrant catkins, the Arabs distil their celebrated caluf water, which they use as a cooling liquor or as a febrifuge.—*Lightfoot.*

12. Fraxinus. *F. excelsior.*

*Ang.* Common ash tree.

*Gen. Desc.* Calyx none, or with four divisions. Blossom none, or with four petals. Female and hermaphrodite flowers. Pist. one, caps. two-celled, leaf-like upwards, compressed; one cell barren. Seed spear-shaped.

*Spec. Desc.* Leaf is serrated ; flowers without petals. *Leaves* opposite, on leaf stalks ; (sometimes simple) *leafits* sitting, four or five pair with an odd one. Some trees produce only male flowers, some only female, and some hermaphrodite. *In woods and hedge-rows.* Bloss. March to May.

*Use.* The bark and seeds are reckoned diuretic.—*Lightfoot.* An infusion of the leaves, from half an ounce to an ounce ounce and a half is a very good *purge;* and a decoction of two drachms of the *bark*, or of six drachms of the *leaves*, has been used to cure agues. The seeds are acrid and bitter. The *bark* is used for tanning calf-skin. A slight infusion of it, viewed between the eye and the light, appears of a pale yellowish colour, but if looked down on, or placed between the eye and an opake object, it is blue. Acids destroy this blue colour, and alkalis recover it again. The *ashes of the wood* afford very good potash. The wood has the singular advantage of being nearly as good when young as when old. It is hard and tough, and much used for tools in husbandry. The roots run near the surface, and extend themselves to a great distance, whence it is destructive to the herbage in upland pastures, but on the margins of ditches or low meadows, the roots act as under-drains, and render the ground hard and firm; but in this case the wood is of little value. It will give a good, though not a beautiful green to cloths that have been blued. In the North of Lancashire the tops of this tree are used in Autumn, when the grass is on the decline, for the food of cattle, which peel off the bark to eat. In very dry summers the leaves are used to feed cows sometimes in Staffordshire. Horses, cows, sheep, and goats eat it, but it spoils the milk of cows, and should not be planted in dairy farms.—*Withering*. In Queen Elizabeth's time, the inhabitants of Colton and Hawksheadfells, remonstrated against the number of forges in the country, because they consumed all the loppings and croppings, the *sole winter food* for cattle.—*Pennant.* From this tree in warm climates exudes the sweet gum, sold as a gentle *cathartic* by the name of manna, though not so abundantly as from the F. ornus.—*Lightfoot.* In the church-yard of Lochaber, Dr. Walker measured the trunk of a dead ash, which, at five feet from the ground, was fifty-eight feet in girth.

### CLASS III. TRIANDRIA.

### Triandria, Monogynia.

1. VALERIANA. *V. officinalis. V. sylvestris. V. Sylvestris major.*

*Ang.* Valerian, great wild valerian.

*Gen. Desc.* Calyx none. Bloss. 1 petal, superior, gibbous at the base on one side. Seed 1, or else a three-celled capsule.

*Spec. Desc. Leaves* winged and toothed. *Blossom,* pink. Upper floral leaves spear-shaped. *Hedges, woods, marshes, commons.* BLOSS. June.

*Use.* It is a *variety* of this species with narrower leaves, which has so great repute as a medicine; it does not exceed two feet in height, and loves dry heaths and pastures. The root has a strong, disagreeble smell, and a warm, bitter, sub acrid taste, both which it communicates to wine, water, or spirit. It possesses *antispasmodic* virtues in an eminent degree.—*Withering.* Its efficacy in *epilepsy* is proved by many instances recorded by a variety of writers; and it has been found highly serviceable in other complaints, termed nervous, particularly those produced by increased mobility or irritability of the nervous system. Bergius states its virtues to be *antispasmodic, diaphoretic, emmenagogue, diuretic, anthelmintic.* Dr. Cullen attests its antispasmodic powers. The root, in substance, is most effectual, and is usually given in powder from a scruple to a drachm.—*Woodville.* It may be taken from half a drachm to two drachms for a dose.—*Withering.* In habitual costiveness it is an excellent medicine, and frequently loosens the bowels when stronger purgatives have failed. Cats are delighted with the smell of the roots, and rats also, insomuch that rat-catchers employ them to draw rats together.—*Withering.* Cows eat the leaves, sheep refuse them.

2. Valeriana. *V. locusta.*

*Ang.* Lamb's lettuce. Corn sallad. Lettuce valerian.

*Gen. Desc. As above.*

*Spec. Desc. Stem* forked. *Leaves* strap-shaped, entire, (in some varieties serrated, or jagged) fringed with white hairs as well as the stem. *Blossom* bluish white. *Common in corn-fields.* Bloss. April and July.

*Use.* The young leaves in spring and autumn are eaten as sallad, and are very little inferior to young lettuce. Cattle eat it.

3. Bryonia. *B. dioica. B. alba. B. aspera. B. alba vulgaris.*

*Ang.* Bryony, red-berried bryony, wild vine.

*Gen. Desc.* Calyx five-toothed; bloss. five divisions; male anthers united at the base. Female, style three-cleft; berry roundish, mostly one-seeded.

*Spec. Desc. Leaves* imperfectly hand-shaped, rough on both sides, with callous points. Male and female flowers on different plants. Barren and fertile plants rarely grow near together. *Blossom* yellow with greenish streaks. *Berry* red. *Seeds* three to six. *Hedges and thickets.* Bloss. May.

*Use.* Both the root and its juice, (which, in spring, if the top top of the root be bared of earth and cut transversely over, is thrown up in abundance for two or three days,—*Lewis*) have a disagreeable smell, and a nauseous, bitter, biting taste, and if applied to the skin they inflame or even vesicate the part. Bergius states this root to be *purgans, hydragoga, emmenagoga, diuretica,* and recommends it in *dropsy* and *asthma.* This powerful and irritating cathartic, now seldom prescribed, is said to be of great efficacy in evacuating serous humours, and has been chiefly employed in hydropical disorders, (two drachms have been given in dropsy with success,—*Lightfoot*). Instances are mentioned of its good effects also in asthma, mania, and epilepsy. In small doses it is said to act as a diuretic and to be resolvent and deobstruent; in powder, from a scruple to a drachm, it is strongly purgative; the juice has a similar effect but more gently. An extract, prepared by water, acts more mildly and with greater safety than the root in substance, and given from half a drachm to one drachm infused in wine, half an ounce is a full dose.—*Withering.* Externally the fresh root has been employed in cataplasms as a resolvent and discutient, also in ischiatic and other rheumatic affections.—*Woodville.* A cold infusion of the root in water is used externally in sciatic pains; a cataplasm of it is a most powerful discutient. A decoction made with one pound of the fresh root is the *best purge for horned cattle.* The active virtues of this plant seem to claim more attention than is now bestowed on it.—*Withering.* Goats eat it. Horses, cows, sheep, and swine refuse it.—*Linn.*

4. Crocus. *C. sativus. C. officinalis. C. officinalis autumnalis.*

*Ang.* Saffron, common, or autumnal saffron.

*Gen. Desc.* Bloss. six equal divisions, summits coiled.

*Spec. Desc. Sheath* one valve rising from the root; tube of the blossom very long. *Blossom* purplish blue; filaments purple; summits deep orange, germen cylindrical. *Cambridgeshire, Essex, and near Derby.* Bloss. August, and September.

*Var. C. vernus, or C. officinalis sylvestris,* spring saffron, or crocus, leaves broader, flat edges. Blossom both yellow and blue. Bloss. March.

*Use.* The summits of the pistils of the *crocus offic.* is the saffron of the shops, carefully collected and moderately dried, in the following manner. In autumn the flowers are gathered in the morning, and the summits with a portion of the style are picked off, and dried by means of a sort of portable kiln, of a peculiar construction, over which which is spread a hair-cloth; this is covered with white paper, on which the summits are placed, two or three inches thick, then covered again with several sheets of paper, over which is laid a blanket, five or six times folded, or a canvas bag filled with straw; on this, when the kiln is heated, a board with a weight on it, is placed, to press the saffron into a cake. For the first hour a pretty strong fire is employed; the saffron is then formed into a cake, which being turned is subjected to the same heat for another hour; it is then turned again; and for twenty-four hours, or till it becomes dry, a gentle heat is applied, turning it every half hour. English saffron is preferred to any imported, and is distinguished by its parts being larger and broader. It affords a beautiful colour to water, wine, or spirit, and gives out the whole of its virtues, and three-fourths of its substance to them. Schroder asserts, that in the quantity of two or three drachms it proves fatal, and it has been said to produce intoxication and madness; it is said to penetrate every part of the body, tinging with yellow the solids and excretions, and to have, even by its odour or effluvia, produced deleterious effects; it has been considered as a most exhilarating cordial, and has been supposed to have much efficacy as an *emmenagogue*, as a *diaphoretic*, and as an *antiseptic*; but modern practice pays no great attention to it, since it has been found to produce no sensible effect, even in very large doses.—*Woodville, Withering, Cullen, &c.* It was held in high estimation by the Hebrews, who called it Carcom; and by the Arabians, by whom it was named Zaffaran. It has a powerful penetrating smell, and a warm, pungent, bitter taste.

5. Iris. *I. pseudacorus, I. palustris, Acorus adulterinus, A. palustris.*

*Ang.* Water flag, yellow flag, flower de luce, yellow water flower de luce, Sedge.

*Gen. Desc.* Bloss. six div. alternate segment bent back as if jointed. Summits petal-like, two-lipped, edges at the base turned in.

*Spec. Desc.* Alternate segment of the blossom smaller than the summit. *Blossom* yellow. *Petals*, the three outer toothed on each side, and streaked with purple. *Germen*, edges furrowed; summits cut into fringed segments at the top. *Valves* of calyx spear-shaped. Flowers three together at the top of the stem; two outer flowers, with each one sheathing valve, the middle flower two. This flower has the appearance of nine petals. *River-banks, marshes, wet meadows.* Bloss. July.

*Use.*

*Use.* The root has an acrid styptic taste, and its juice snuffed up the nostrils, produces a burning in the nose and mouth, with a copious discharge from these organs; hence it is recommended as an *errhine* and *sialagogue.* The root well dried is a powerful *astringent,* and has been successfully used in the cure of diarrhœas; but the fresh root and its juice are strongly *cathartic,* insomuch that 80 drops of the latter given every hour or two *(by Dr. Rutherford, Ed. Med. Ess. vol. v. p.* 94,*)* produced repeated evacuations after the most powerful purgatives had failed, and by its continued use in increased doses, cured a violent dropsy.—*Woodville.* But the degree of its acrimony is so uncertain that it can hardly ever come into general use; the juice expressed from the old roots is the most active. In some cases it is *diuretic.* The fresh roots have been mixed with the food of swine bitten by a mad dog, and they escaped the disease, when others bitten by the same dog, died raving mad.—*Withering.* The root dried has been used instead of galls in making ink, and also in dying black. For the latter purpose it is employed in the island of Jura.—*Pennant.* In Mull and other parts of the Highlands, the root of this plant is used to cure the tooth-ach, or any inflammation of the throat; a piece about the size of a nutmeg is bruised in a mortar with a handful of daisies; the juice is strained through linen, and a tea-spoonful of it poured into each nostril. It is immediately followed by a kind of salivation which often effects a cure, but not without great danger of cold.—*Lightfoot.* The expressed juice is said to be an useful application to serpiginous eruptions and scrophulous tumors.—*Murray Ap. Med. vol. v. p.* 277. Goats eat the fresh leaves; cows, horses, and swine refuse them; cows will eat them when dry. The juice of this root as well as of the *Iris fœtida, stinking flag, or stinking gladwyn,* have been used to excite sneezing, but it is a dangerous practice, and sometimes produces violent convulsions.

6. Scirpus. *S. lacustris.*

*Ang.* Bull rush, club grass.

*Gen. Desc.* Husks chaff-like, tiled on every side; bloss 0, seed one; three-cornered, woolly.

*Spec. Desc.* *Straw* cylindrical, naked; spikes several, egg-shaped, on fruit stalks, terminating; calyx fringed three-cleft. *Rivers, pools, fens.* Bloss. July, August.

*Use.* When fodder is exhausted, cattle will live upon it. Cottages are thatched, and pack saddles stuffed with it. Chair-bottoms are also made of it; cut at one year old it makes the fine bottoms; at two the coarser; and at a greater age, mixed with the leaves of Iris pseudacoras, the coarsest bottoms. Mats are also made of it, either alone or thus mixed. Goats and swine eat it. Cows and sheep refuse it.

7. SCIRPUS. *S. maritimus.*
*Ang.* Salt marsh club grass.
*Gen. Desc.* *As above.*
*Spec. Desc.* *Straw* three-cornered; panicle close, leafy; scales of the spickets three-cleft, middle segment awl-shaped. *Leaves* stiff, sharp at the edges. *Panicle* sometimes branched, sometimes simple. *Spikes* oblong, rusty iron coloured. *Seeds* the same colour, egg shaped, compressed, tapering. *Sea-coast.* BLOSS. August.
*Use.* The roots dried and ground to powder have been used as a substitute for flour in times of scarcity. Cows eat it.

8. CYPERUS. *C. longus, C. odoratus radice longâ.*
*Ang.* Sweet cyperus, English galingale, long cyperus.
*Gen. Desc.* *Husks* chaff like; tiled in two rows. Bloss. 0. Seed 1, naked.
*Spec. Desc.* Straw three cornered, leafy; *Umbel* leafy more than doubly compound. *Fruitstalks* naked, sometimes twelve or thirteen, forming a sort of umbel. *Spikes* alternate; *little spikes* slender, chesnut coloured. BLOSS. May.
*Use.* The root is agreeably aromatic to the smell; warm and bitter to the taste. In modern practice it is disregarded; but perhaps it is not inferior to some of the most costly medicines brought from abroad.—*Withering.* The roots are carminative and attenuant; they promote the menses, and are useful in chronic cases arising from obstructions of the viscera.—*Hill.*

[ To be continued. ]

